# Contralateral Hip Abductor Muscle Strength Associated with Comfort of Getting into and out of the Car after Total Hip Arthroplasty

**DOI:** 10.3390/jcm12175515

**Published:** 2023-08-25

**Authors:** Tetsunari Harada, Satoshi Hamai, Daisuke Hara, Tsutomu Fujita, Daisuke Fujiyoshi, Shinya Kawahara, Ryosuke Yamaguchi, Kenichi Kawaguchi, Yasuharu Nakashima

**Affiliations:** 1 Department of Orthopaedic Surgery, Faculty of Medical Sciences, Kyushu University, 3-1-1 Maidashi, Higashi-ku, Fukuoka 812-8582, Japan; life_rageblue_rain@yahoo.co.jp (T.H.); hara.daisuke.605@m.kyushu-u.ac.jp (D.H.); kawahara.shinya.310@m.kyushu-u.ac.jp (S.K.); yamaguchi.ryosuke.183@m.kyushu-u.ac.jp (R.Y.); kawaguchi.kenichi.241@m.kyushu-u.ac.jp (K.K.); nakashima.yasuharu.453@m.kyushu-u.ac.jp (Y.N.); 2 Department of Rehabilitation Medicine, Kyushu University Hospital, 3-1-1 Maidashi, Higashi-ku, Fukuoka 812-8582, Japan; paradigm.shift.2106@gmail.com (T.F.); fujiyoshi.daisuke.770@m.kyushu-u.ac.jp (D.F.)

**Keywords:** total hip arthroplasty, car, muscle strength, rehabilitation

## Abstract

There are no studies that have investigated the characteristics of car use across THA patients, including those who do not drive. This study aimed to evaluate, in THA patients, (1) postoperative car usage, (2) comfort while entering and exiting a car, and (3) whether lower limb muscle strength affects action comfort. One hundred seventy-two post-THA patients completed the questionnaire in 2020, along with assessments of hip abductor and knee extensor muscle strength before surgery and at discharge. Patients whose overall comfort level was judged as comfortable were defined as the comfort group; others were placed in the discomfort group. Of the 172 patients, 161 reported car usage at a mean of 5.6 years after THA. Of these, 114 and 47 patients were placed in the comfort and discomfort groups, respectively. Patients in the discomfort group were three times more likely to experience discomfort using the contralateral side door than the surgical side door, and about twice as many patients experienced discomfort when entering as when exiting. Lower preoperative contralateral hip abductor muscle strength was the only independent predictor for discomfort. The take-home messages were that prevention of contralateral-side weakness may improve comfort during the action after THA.

## 1. Introduction

Total hip arthroplasty (THA) is considered one of the most successful orthopedic procedures performed on patients with osteoarthritis (OA) [1]. Fujita et al. [2] report that improved postoperative activities of daily living (ADLs) and sports are important to improve satisfaction after THA. The ability to successfully perform common ADLs is important for safe mobility, social participation, and ultimately quality of life.

In modern society, car usage is one of the most important ADLs. Shiomoto et al. [3] report that getting into and out of a car is significantly correlated with the perception of a natural joint after THA. Previous studies on car use after THA have focused on patients’ return to driving [4]. To our knowledge, there are no studies that have investigated the characteristics of car use across THA patients, including those who do not drive.

Therefore, we performed a retrospective study, in patients with OA who underwent THA to assess: (1) How many of them use a car, and the delay to using one post-surgery, (2) how comfortable they were while getting into and out of a car, and (3) what factors affected their comfort in getting into and out of a car after THA.

## 2. Materials and Methods

### 2.1. Patients

**Figure 1:**
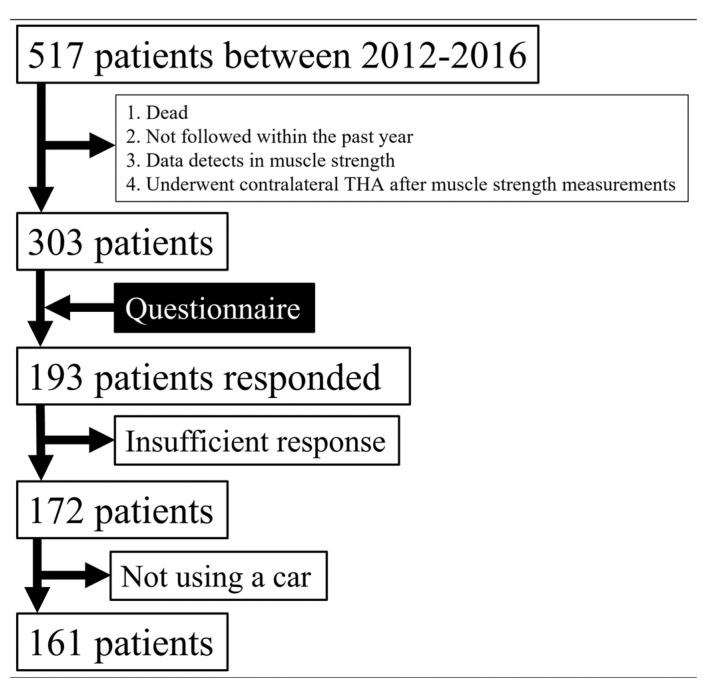
Schematic representation of study cohort development. THA, total hip arthroplasty.

After approval from the Institutional Review Board (IRB number: 21142-00), we retrospectively reviewed the clinical course of 517 patients who underwent THA for OA between January 2012 and December 2016. All THAs were performed using a posterolateral approach, with a uniform protocol for postoperative rehabilitation [5]. Among them, 303 patients satisfied the following inclusion criteria: (1) at least one year elapsed since surgery, (2) evaluation by a surgeon within the past year, (3) no defects in muscle strength data, and (4) no contralateral THA after muscle strength measurements. A total of 303 THA patients received the study questionnaire in the mail, of which 193 (63.7%) returned the questionnaire with written informed consent. Of the respondents, 172 (56.1%) provided satisfactory responses and comprised the final study cohort (Figure 1). All demographic and clinicopathological information was obtained from the patients’ medical records. Data were handled following the ethical standards of the Declaration of Helsinki. The study cohort was composed of 26 males and 146 females. The mean age at surgery, body mass index (BMI), and follow-up duration were 64.8 ± 10.1 years, 24.0 ± 3.5 kg/m^2^, and 66.8 ± 15.4 months, respectively.

### 2.2. Questionnaire

All participants were sent a 9-item questionnaire to collect the following information in 2020 (Table 1): (1) Whether the patient used a car, (2) whether the patient drove, (3) the timing of returning to car usage, (4) comfort level while getting into and out of a car, (5) the reasons for feeling uncomfortable when getting into and out of a car, and (6) the door side on which getting into and out of a car was uncomfortable, (7) which side was more uncomfortable, (8) satisfaction with pain level^8^, and (9) satisfaction with function [6]. Questions numbered 1 through 7 were created specifically for this study, while questions numbered 8 and 9 were adapted from an existing questionnaire [6]. Comfort level of getting into and out of a car showed positive correlations (Spearman’s correlation coefficient ρ = 0.47, *p* < 0.001) with functional satisfaction, suggesting convergent construct validity.

### 2.3. Muscle Strength Measurement

**Figure 2:**
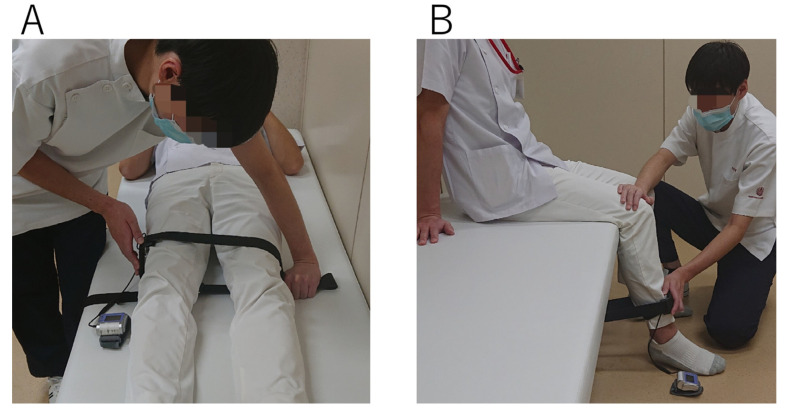
Muscle strength measurement. (**A**) Hip abductor measurement; patients were supine with the hip and knee in a neutral position; a force sensor (μ-Tas F1) was placed 5 cm proximal to the lateral epicondyle of the femur. (**B**) Knee extensor measurement; patients were seated with straps across the waist and thighs.

All patients underwent an assessment of maximal voluntary isometric muscle strength in concentric conditions using the hand-held dynamometer (HHD) (μ-Tas F1; ANIMA Inc., Tokyo, Japan) before surgery and at discharge (Figure 2). Hip abductor and knee extensor strengths were quantified by well-trained physiotherapists with more than a year of experience at the moment of testing [7]. Two trials were performed after one practice in all examinations, with the highest peak torque (Nm/kg) used for the analysis.

### 2.4. Statistics

Patients who responded comfortable were defined as the comfort group, and those who responded “somewhat uncomfortable”, “very uncomfortable”, or “impossible” were defined as the discomfort group. Patient characteristics, postoperative satisfaction, and muscle strengths were compared between comfort and discomfort groups and between surgical and contralateral side using Student’s *t*-test. Logistic regression analysis was performed to identify predictors associated with comfort getting into and out of a car after THA. Before analysis, we selected the predictive factors with a *p*-value of <0.05 in univariate analyses [age, sex, BMI, surgical side (unilateral or bilateral), contralateral hip (normal, OA or THA), and muscle strengths before THA and at discharge], and then a stepwise method was conducted to select the factors for exclusion of confounding factors. Logistic regression analysis was conducted using the factors selected using the stepwise method. All statistical analyses were performed using JMP^®^ Pro 16 (SAS Institute Inc., Cary, NC, USA) with a significance level established at 0.05. Power analyses showed that the combined sample size of the two groups was 61, which provided 80% statistical power to detect the difference in muscle torque of HHD between the two groups [7].

## 3. Results

### 3.1. Car User Group

Of the 172 patients, 161 (24 males and 137 females) used a car after THA (Figure 1). The mean age at surgery, BMI, and follow-up duration were 65.0 ± 10.1 years, 24.0 ± 3.5 kg/m^2^, and 66.7 ± 15.3 months, respectively. Of the 161 patients, 101 were the driver, while the remainder were passengers in the front or rear seats. In total, 87 (54.0%) resumed driving or riding as a passenger within 1 month after surgery, 52 (32.3%) within 1 to 3 months, 9 (5.6%) within 3 to 6 months, 11 (6.8%) within 6 to 12 months, and 2 (1%) after 12 months. Hip abductor strength (surgical/contralateral side) before surgery and at discharge (a mean of 3.3 weeks) averaged 0.44/0.57 Nm/kg and 0.47/0.61 Nm/kg, respectively; hip abductor strength of the surgical side was significantly lower than for the contralateral side (*p* < 0.0001 and 0.0001, respectively). Knee extensor strength (surgical/contralateral side) averaged 0.74/0.94 Nm/kg and 0.75/0.99 Nm/kg, respectively; knee extensor strength of the surgical side was significantly lower than for the contralateral side (*p* < 0.0001 and 0.0001). Hip abductor strength of the surgical and contralateral sides improved significantly between pre- and postoperative assessments (*p* = 0.036 and 0.039), while there was no significant difference in knee extensor strength (*p* = 0.681 and 0.175). The hip abductor and knee extensor strength of the surgical side before surgery and at discharge showed positive correlations (Spearman’s correlation coefficient ρ = 0.74, 0.71, 0.73, and 0.70, *p* < 0.0001, 0.0001, 0.0001, and 0.0001) with the contralateral side.

### 3.2. Comfort and Discomfort Groups

Of the 161 patients, 114 (70.8%) were placed in the comfort group (Table 2). There were no significant differences in age, sex, BMI, follow-up duration, hospitalization duration, surgical side (unilateral/bilateral and left/right), and contralateral hip (normal/osteoarthritis/THA) between the comfort and discomfort groups (*p* > 0.05; Table 2). In the discomfort group, 20 patients (18 right and 2 left THA patients), 14 patients (6 right and 8 left THA patients), and 13 patients (6 right and 7 left THA patients) felt more uncomfortable using left, right, and both door sides, respectively; 26 patients felt more uncomfortable using the door of the contralateral side, about three-fold more than the 8 patients who felt more uncomfortable using the door of the surgical side. In addition, 28 patients experienced more discomfort when getting into a car, almost twice as many as the 15 patients who experienced discomfort when getting out (Table 3). The main reason for discomfort was functional limitations of the surgical side, followed by pain (Table 3). The discomfort group contained significantly more females and were less satisfied with pain and function (*p* < 0.05; Table 4).

The comfort and discomfort groups had significantly lower hip abductor and knee extensor strength on the surgical side than on the contralateral side before surgery and at discharge (comfort group; *p* < 0.0001, 0.0001, 0.0001, and 0.0001; discomfort group; *p* = 0.001, 0.027, 0.0003, and 0.0004). The discomfort group had significantly lower contralateral hip abductor strength and contralateral knee extensor strength before surgery and at discharge than the comfort group (*p* = 0.010, 0.013, 0.021, and 0.042; Table 4), while there were no significant differences in hip abductor and knee extensor strength of the surgical side (*p* > 0.05; Table 4). There were no significant differences in the changes of surgical and contralateral hip abductor strength and knee extensor strength from preoperative to discharge between comfort and discomfort groups. (*p* = 0.334, 0.569, 0.501, and 0.419; Table 4).

### 3.3. Predictors Influencing Comfortable Getting into and Out of a Car

Univariate analysis showed that sex and preoperative hip abductor and knee extensor strength of the contralateral side were significantly correlated with comfort getting into and out of a car after THA (*p* = 0.037, 0.008, and 0.009; Table 5).

Multivariate analysis using significant factors (sex and contralateral hip abductor and contralateral knee extensor strength before surgery; Table 5) in univariate analysis showed that lower preoperative contralateral hip abductor strength was the only significant predictor of discomfort when getting into and out of a car (*p* < 0.05; Table 6). Preoperative contralateral hip abductor strength (AUC, 0.631; *p* = 0.008; threshold ≥ 0.49; sensitivity of 72.1%, specificity of 50.0%) showed predictive accuracy for comfort in getting in and out of a car.

## 4. Discussion

This study is the first to report on the characteristics of getting into and out of a car after THA. Approximately 94% of patients used a car at mid-term after THA. Of them, 71% of patients felt comfortable getting into and out of a car, with the preoperative contralateral hip abductor muscle strength being a significant predictor. A preoperative hip abductor strength of ≥0.49 Nm/kg was required to comfortably get into and out of a car after THA.

**Figure 3:**
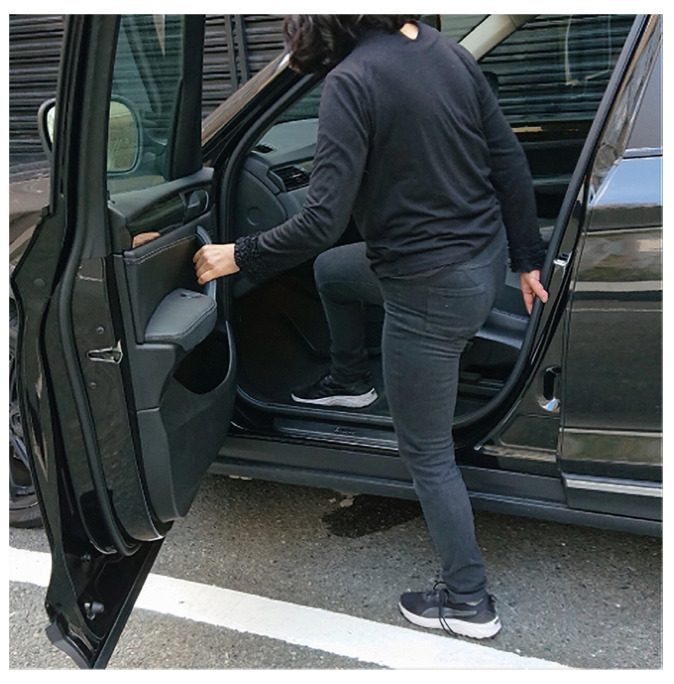
Actual scene when right THA patient gets into the passenger seat of a right-hand car using the contralateral side door and the contralateral side as the pivot limb.

Twenty-nine percent of patients who received primary THA reported feeling uncomfortable getting into and out of a car. Patients in the discomfort group were about three-fold more likely to experience discomfort when using the door on the contralateral side than the door on the surgical side, and about twice as many patients experienced discomfort when getting into a car as when getting out. These findings may indicate that this cohort of patients were more likely to feel discomfort when using the contralateral side as the pivot limb than the surgical side and found getting into a car more uncomfortable than getting out of a car (Figure 3). The primary reason for discomfort was functional limitations of the surgical side, followed by pain. In addition, the patients had significantly lower pain and functional satisfaction compared to the comfortable group. Previous studies have reported the importance of functional improvement and pain relief in improving patients’ ADLs and sports participation after THA [2,8,9]. This study also emphasized the importance of functional improvement and pain relief. The significantly higher proportion of females in the discomfort group was consistent with previous reports that demonstrated the association between sex and clinical outcomes after THA [2,8].

Patients in this study, with a mean age of 65 years, had 0.57 Nm/kg and 0.61 Nm/kg of isometric hip abduction muscle strength on the contralateral side before surgery and at discharge (mean 3.3 weeks postoperatively), respectively, which was comparable to the isometric hip abduction muscle strength (0.57 Nm/kg) of healthy participants of a similar age [10]. Isometric hip abduction muscle strength on the surgical side was 0.44 Nm/kg and 0.47 Nm/kg before surgery and at discharge, respectively. Fukushi et al. [7] reported isometric hip abduction muscle strength on the surgical side relative to the contralateral side, preoperatively and 1 month postoperatively in THA patients with a mean age of 66 years to be 78% and 77%, respectively. These values are comparable to the 77% and 77% found in the present study, indicating that the current muscle strength data were adequate. Lower hip abductor muscle strength on the contralateral side preoperatively was a predictor of the perceived feeling of discomfort concerning getting into and out of a car in the mid-term after THA. Preoperative hip abductor strength of ≥0.49 Nm/kg was required to comfortably get into and out of a car after THA, which can be used as a preoperative screening. The discomfort group had lower contralateral hip abductor strength than the comfort group, and the change in muscle strength between pre- and postoperative assessments was not significantly different between comfort and discomfort groups, suggesting that lower hip abductor muscle strength of the contralateral side persisted from preoperative to postoperative assessment. Although the patients subjectively felt that discomfort during getting in and out of a car was caused by the functional limitations of the surgical side, it was objectively due to contralateral hip abductor muscle weakness, supported by a correlation between the hip abductor muscle strength of the surgical and contralateral side. These results suggest that it is important to prevent contralateral muscle weakness prior to THA. Omori et al. [11] reported that clinical outcome after THA is influenced by preoperative contralateral limb muscle strength, and the contralateral limb muscle weakness persists postoperatively after surgery, which is consistent with the findings of this study. Patients with osteoarthritis of the hip first experience muscle weakness of the involved side and impaired balance and muscle function. With worsening osteoarthritis, the patients experience overall disuse and contralateral weakness [12]. Surgery before contralateral lower-limb function worsens may lead to more comfortable use of a car after THA. Furthermore, Moore et al. [13] report that single-legged standing with contralateral lower extremity motion results in higher hip abductor muscle activation of the pivot limb. Thus, the hip abductor muscle strength of the pivot leg plays a significant role in getting into and out of a car, and a lower hip abductor muscle strength of the contralateral side may influence the discomfort felt by many patients when getting into and out of a car with the contralateral side as the pivot leg. Meanwhile, our recent study showed, in a 3D dynamic analysis using a motion capture method, that getting into and out of a car with the contralateral side as the pivot hip is more similar to the dynamic of a healthy patient than using the surgical side as pivot hip; furthermore, that strengthening of the abductor and extensor muscles of the surgical hip is important for a balanced motion when using the surgical hip as the pivot limb [14]. This difference in findings may be explained by the fact that the present study included patients with OA and THA on the contralateral side and patients who required aids for walking, unlike the patients included in the 3D dynamic analysis.

The current study has several limitations. Unreturned and missing questionnaire data impacted the study’s sample size. However, the overall 56% response rate was comparable with previous studies [2,3]. In addition, the physical function data at the time of the questionnaire was not analyzed. Thus, the relationship between muscle strength and comfort in getting into and out of a car in the mid-term postoperative period was not assessed; however, this study was able to show that preoperative physical function predicted motion comfort in the mid-term postoperative period. This study’s analysis did not control for discomfort caused by the steering wheel, so the results may be different in Western countries, where the driver’s seat is primarily on the left side. However, fewer patients felt uncomfortable getting into and out of a car using the right door than the left door, and there were no patients who responded that the reason for their discomfort in getting into and out of the driver’s seat was the presence of a steering wheel.

## 5. Conclusions

Approximately 94% of the patients were using a car at mid-term after THA, and 29% of them felt discomfort while getting into and out of a car, especially when using the contralateral side as the pivot limb and during entry. Functional limitations of the surgical side were primarily attributed as the reason for this subjective impression. However, the predictor of discomfort concerning getting into and out of a car was a preoperative decline in contralateral hip abduction muscle strength, and prevention of this weakness prior to surgery was found to be important. Preoperative hip abductor strength of ≥0.49 Nm/kg was required to comfortably get into and out of a car after THA, which can be used as a preoperative screening.

## Figures and Tables

**Table 1:** Nine-item questionnaire.

Question	Possible Answer
Do you use a car?	Yes No
Do you drive a car?	Yes No
When did you return to using a car?	Within 1 month 1–3 months 3–6 months 6–12 months After 12 months
How comfortable are you getting into and out of a car?	Comfortable Somewhat uncomfortable Very uncomfortable Impossible
Why is getting into and out of a car uncomfortable?	Free description
Which door side is uncomfortable for you to get into and out of a car?	Left door Right door Both doors None
Which action is uncomfortable for you to get into or out of a car?	Getting into a car Getting out of a car Both actions None
How satisfied are you with the results of your surgery for improving your pain?	Very satisfied Somewhat satisfied Somewhat dissatisfied Very dissatisfied
How satisfied are you with the results of surgery for improving your ability to do recreational activities?	Very satisfied Somewhat satisfied Somewhat dissatisfied Very dissatisfied

**Table 2:** Comfort level of getting into and out of a car.

Comfort Level	*n* = 161; *n* (%)
Comfortable	114 (70.8)
Somewhat uncomfortable	30 (18.6)
Very uncomfortable	17 (10.6)
Impossible	0 (0)

**Table 3:** Discomfort group characteristics.

Question	*n* = 47; *n* (%)
Reasons for discomfort getting into and out of a car	
Functional limitations	31 (66.0)
Pain	11 (23.4)
Anxiety (falls and dislocations)	4 (8.5)
Physical factor (small height)	1 (2.1)
Door side where getting into and out of a car is uncomfortable	
Door of surgical side	8 (5.0)
Door of contralateral side	26 (16.1)
Both door side	13 (8.1)
None	114 (70.8)
Uncomfortable action	
Getting into a car	28 (17.4)
Getting out of a car	15 (9.3)
Both actions	9 (5.6)
None	109 (67.7)

**Table 4:** Comparison of demographics, satisfaction, and muscle strength data.

Parameters	All Patients (*n* = 161)	Comfort (*n* = 114)	Discomfort (*n* = 47)	*p* Value *
Age (y)	65.0 ± 10.1	64.1 ± 9.5	67.2 ± 11.3	0.084
Male/female, *n* (%)	24/137 (14.9/85.1)	21/93 (18.4/81.6)	3/44 (6.4/93.6)	0.037 *
BMI (kg/m^2^)	24.0 ± 3.5	23.9 ± 3.3	24.2 ± 4.0	0.681
Follow-up duration (months)	66.7 ± 15.3	67.7 ± 14.8	64.4 ± 16.6	0.215
Hospitalization duration (weeks)	3.3 ± 1.0	3.2 ± 0.8	3.6 ± 1.2	0.424
Surgical side (unilateral/bilateral), *n* (%)	125/36 (77.6/22.4)	90/24 (79.0/21.0)	35/12 (74.5/25.5)	0.535
Surgical side (left/right), *n* (%)	74/87 (46.0/54.0)	57/57 (50.0/50.0)	17/30 (36.2/63.8)	0.109
Contralateral hip (normal/OA/THA), *n* (%)	92/33/36 (57.1/20.5/22.4)	67/23/24 (58.8/20.2/21.0)	25/10/12 (53.2/21.3/25.5)	0.779
Satisfaction-pain				
(very satisfied/somewhat satisfied/somewhat dissatisfied/very dissatisfied), *n* (%)	124/34/3/0 (77.0/21.1/1.9/0)	96/17/1/0 (84.2/14.9/0.9/0)	28/17/2/0 (59.6/36.2/4.2/0)	0.004 *
Satisfaction-function				
(very satisfied/somewhat satisfied/somewhat dissatisfied/very dissatisfied), *n* (%)	80/77/3/0 (50.0/48.1/1.9/0)	69/44/1/0 (60.5/38.6/0.9/0)	11/33/2/0 (23.9/71.7/4.4/0)	<0.0001 *
Muscle torque before surgery (Nm/kg)				
Hip abduction of surgical side	0.44 ± 0.16	0.44 ± 0.18	0.42 ± 0.11	0.524
Hip abduction of contralateral side	0.57 ± 0.18	0.59 ± 0.19	0.51 ± 0.14	0.010 *
Knee extension of surgical side	0.74 ± 0.33	0.75 ± 0.34	0.71 ± 0.30	0.534
Knee extension of contralateral side	0.94 ± 0.35	0.99 ± 0.37	0.83 ± 0.27	0.013 *
Muscle torque at discharge (Nm/kg)				
Hip abduction of surgical side	0.47 ± 0.16	0.49 ± 0.16	0.44 ± 0.16	0.109
Hip abduction of contralateral side	0.61 ± 0.17	0.63 ± 0.18	0.56 ± 0.14	0.021 *
Knee extension of surgical side	0.75 ± 0.25	0.77 ± 0.25	0.70 ± 0.24	0.110
Knee extension of contralateral side	0.99 ± 0.34	1.03 ± 0.35	0.91 ± 0.30	0.042 *
Change in muscle torque from preoperative to discharge (Nm/kg)				
Hip abduction of surgical side	0.04 ± 0.17	0.05 ± 0.16	0.02 ± 0.19	0.334
Hip abduction of contralateral side	0.04 ± 0.14	0.04 ± 0.15	0.05 ± 0.12	0.569
Knee extension of surgical side	0.01 ± 0.24	0.02 ± 0.26	−0.01 ± 0.21	0.501
Knee extension of contralateral side	0.05 ± 0.25	0.04 ± 0.24	0.08 ± 0.27	0.419

Continuous values are expressed as mean ± standard deviation. OA, osteoarthritis; THA, total hip arthroplasty. * *p* < 0.05 for the comparison between comfort group and discomfort group.

**Table 5:** Univariate analysis of predictors influencing comfortable getting into and out of a car.

Variables	Estimate (Standard Error)	95% CI	Negative Factor	*p* Value
Age	−0.03 (1.20)	−0.07, 0.004		0.081
Sex (female)	−0.60 (0.32)	−1.34, −0.03	Female sex	0.037 *
BMI	−0.002 (0.05)	−0.12, 0.08		0.680
Surgical side (unilateral)	0.13 (0.20)	−0.28, 0.52		0.539
Surgical side (right)	−0.28 (0.18)	−0.64, 0.06		0.107
Preoperative hip abduction muscle torque of surgical side	0.69 (1.13)	−1.50, 2.97		0.538
Preoperative hip abduction muscle torque of contralateral side	2.82 (1.12)	0.70, 5.13	Lower muscle torque	0.008 *
Preoperative knee extension muscle torque of surgical side	0.35 (0.56)	−0.71, 1.50		0.526
Preoperative knee extension muscle torque of contralateral side	1.49 (0.61)	0.35, 2.75	Lower muscle torque	0.009 *

* indicates *p* < 0.05.

**Table 6:** Multivariate analysis of predictors influencing comfort getting into and out of a car.

Variables	Estimate (Standard Error)	95% CI	Negative Factor	*p* Value
Sex (female)	−0.59 (0.39)	−1.54, 0.08	Female sex	0.131
Preoperative hip abduction muscle torque of contralateral side	2.35 (1.18)	0.10, 4.74	Lower muscle torque	0.046 *

* indicates *p* < 0.05.

## Data Availability

The data presented in this study are available on request from the corresponding author. The data are not publicly available due to privacy.
